# The influence of alcohol consumption on sickness presenteeism and impaired daily activities. The WIRUS screening study

**DOI:** 10.1371/journal.pone.0186503

**Published:** 2017-10-17

**Authors:** Randi Wågø Aas, Lise Haveraaen, Hildegunn Sagvaag, Mikkel Magnus Thørrisen

**Affiliations:** 1 Department of Health Studies, University of Stavanger, Stavanger, Norway; 2 Presenter - Making Sense of Science, Stavanger, Norway; 3 Department of Occupational Therapy, Prosthetics and Orthotics, Oslo and Akershus University College of Applied Sciences, Oslo, Norway; California Pacific Medical Center Research Institute, UNITED STATES

## Abstract

**Background:**

Alcohol use is a global health issue and may influence activity performance in a variety of domains, including the occupational and domestic spheres. The aim of the study was to examine the influence of annual drinking frequency and binge drinking (≥6 units at one occasion) on activity impairments both at work (sickness presenteeism) and outside the workplace.

**Methods:**

Employees (n = 3278), recruited from 14 Norwegian private and public companies, responded to a questionnaire containing questions from the Alcohol Use Disorders Identification Test (AUDIT) and the Workplace Productivity and Activity Impairment questionnaire (WPAI).

**Results:**

Multiple hierarchical regression analyses revealed that binge drinking was associated with both sickness presenteeism and impaired daily activities, even after controlling for gender, age, educational level, living status and employment sector. Annual drinking frequency was associated with impaired daily activities, but not sickness presenteeism.

**Conclusions:**

Binge drinking seems to have a stronger influence on activity performance both at work and outside the workplace than drinking frequency. Interventions targeting alcohol consumption should benefit from focusing on binge drinking behavior.

## Introduction

Alcohol use constitutes a global health issue. Harmful use of alcohol has been found to be involved in more than 200 different injury and disease conditions [1]. The World Health Organization estimates that 3.3 million annual deaths worldwide; i.e., 5.9% of all global mortality are related to alcohol use [2]. Alcohol consumption levels tend to be highest in the developed world, and alcohol is the most used psychoactive substance in the workforce [3]. Studies have demonstrated that between 10 and 35% of employees can be characterized as risky drinkers [4], i.e., that they have a pattern of alcohol consumption that increases the risk of social, legal, medical, occupational, domestic, and economical problems [5].

Alcohol consumption may influence activity performance in a variety of domains, including the occupational and domestic spheres. In his general model of employee substance use and productivity outcomes, Frone [3] proposes that both on-the-job and off-the-job substance use may lead to impaired performance outcomes. Furthermore, a recent systematic review reported that alcohol consumption is associated with both short- and long-term sickness absence [6]. Reporting to work and performing sub-optimally due to alcohol use, however, has received somewhat less attention in the research literature. This phenomenon, reduced on-the-job productivity, is termed sickness presenteeism. In a longitudinal study, Kirkham and colleagues [7] found that alcohol was associated with a higher number of presenteeism days among both younger and older workers. Similarly, others have discovered positive relationships between drinking behavior and the frequency of reported work problems [8] as well as alcohol consumption and productivity loss [9]. Moreover, sickness presenteeism has been found to be a risk factor for future sickness absence [10].

Alcohol consumption are often associated with impaired daily activities, such as difficulties in carrying out daily routines [11] and mobility problems [12]. Difficulties in economic self-sufficiency (inadequate access to financial resources to support everyday life), restriction of participation in activities associated with leading a meaningful life, and impaired social relationships have also been associated with alcohol consumption [13].

Different drinking patterns can have dissimilar effects on outcome measures. One may distinguish between (a) drinking frequency, i.e., the typical frequency of drinking in a given period of time, and (b) episodic heavy drinking (binge drinking). Binge drinking is often operationalized as consuming five drinks or more on one occasion [14, 15]. However, the Alcohol Use Disorders Identification Test defines binge drinking as six or more alcohol units on a single occasion [5].

In line with Bacharach and colleagues [16], it may be reasonable to assume that impairment-producing episodes of binge drinking would be more predictive of both sickness presenteeism and impaired daily activities than drinking frequency. Effects captured by drinking frequency may be linked to rather long-term ill-health consequences while binge drinking tends to have explicit short-term impairment-related consequences (e.g., hangover symptoms) [17].

The present study was conducted in Norway, a country in which alcohol is a legal and widely used drug. Traditionally, Norway has been characterized as a spirit-drinking country with binge drinking during the weekends and abstinence during weekdays, i.e., a dry drinking culture [18]. However, it has been emphasized that the Norwegian drinking culture has developed during the last decades in the direction of more drinking during weekdays in addition to weekend binge drinking [19]. Nevertheless, Norwegian youths are consuming less alcohol than most of their Western counterparts [20], and alcohol use per person per year in the general Norwegian population (7.7 litres) is somewhat lower than in the rest of Europe (10.9 litres) and in the United States (9.2 litres) [2].

Based on a public health perspective and justified by the total consumption model [21], Norway has restrictive alcohol policies regulated by means of a licence system, alcohol sale monopoly, advertising ban, age limits and taxation on products containing alcohol [20]. Use of alcohol at work is forbidden and infringement may result in resignation. Scandinavian studies on alcohol consumption in the working community have primarily focused on drinking outside the workplace [22]. Although representing a quite uninvestigated issue in Norwegian studies, alcohol-related sickness presenteeism has, in a recent study [23], been reported by 11.0% of employees.

Knowledge on the relationship between alcohol consumption on one hand and sickness presenteeism and impaired daily activities on the other, is limited within working populations that are not in clinical treatment for alcohol abuse or -dependence. To be able to provide early identification and public health programs targeting risky drinking, such knowledge might be crucial. Moreover, there seems to be a shortage of studies that have explored and compared activity restrictions both within and outside the workplace.

The aim of the present study was therefore to explore the influence of annual drinking frequency and binge drinking on sickness presenteeism and impaired daily activities in a sample of Norwegian employees.

## Materials and methods

### Design

This study is part of the Norwegian national WIRUS project (Workplace Interventions preventing Risky Use of alcohol and Sick leave), where one of the studies are the WIRUS-Screening study. Other results from WIRUS are published elsewhere [24]. The study was designed as a cross-sectional study among private (n = 5) and public (n = 9) companies, employing a total of 14,353 individuals.

### Sample

The employees were invited to participate in a web-based alcohol screening study, where they answered questionnaires designed to measure alcohol consumption, sickness presenteeism and impaired daily activities. A total of 4,275 employees (29.8%) responded to the questionnaire. However, 997 employees were excluded because of missing values on key variables or as a result of being abstainers, leaving a final sample of 3,278 individuals. Characteristics of the study sample, the invited sample and the Norwegian workforce are presented in Table 1.

**Table 1:** Study sample, invited sample and national workforce characteristics.

Variable	Study sample % (n)	Invited sample % (n)	Difference % (*p-value*)^a^	Norwegian workforce %^b^
**Gender**			1.6 (.081)	
Male	32.6 (1067)	34.2 (4908)		52.7
Female	67.4 (2211)	65.8 (9445)		47.3
**Age**			4.0 (< .001)	
≤ 39	31.5 (1032)	35.5 (5102)		45.0
≥ 40	68.5 (2246)	64.5 (9251)		55.0
**Educational level**				
Primary/lower secondary	2.5 (81)	-		16.3
Upper secondary	22.2 (728)	-		42.3
University/college	75.3 (2469)	-		41.4
**Living status**				
Living alone	13.7 (448)	-		-
Living with others	86.3 (2830)	-		-
**Employment sector**				
Private	10.0 (328)	-		-
Public	90.0 (2950)	-		-
**Industry**				
Transport	1.8 (60)	-		-
Production	5.6 (184)	-		-
Publ. administration	75.3 (2468)	-		-
Health care	16.5 (542)	-		-
Hotel/restaurant	0.7 (24)	-		-

^a^Difference between study sample and invited sample.

^b^Characteristics of the Norwegian national workforce in 2016, obtained from Statistics Norway (http://www.ssb.no)

The study sample consisted of 32.6% males and 67.4% females. 68.5% of employees were aged ≥40 and 75.3% had completed a university or college education. 10.0% of the respondents were employed within the five private sector companies (production, transport, hotel/restaurant and health care), while 90.0% were employed within the nine public sector companies (public administration and health care).

### Alcohol measures

Two questions were used to measure alcohol consumption. Both items were taken from the Norwegian translation of the Alcohol Use Disorders Identification Test (AUDIT), developed by the World Health Organization [5]. Annual drinking frequency (AUDIT 1), was measured by one item: "How often, during the last year, did you have a drink containing alcohol?". Answers were scored on a five-point Likert scale ranging from "never", "monthly or less", "two or four times a month", "two to three times a week" to "four or more times a week". Employees who responded "never" on the AUDIT-1 were treated as abstainers and consequently excluded from the final sample. Hence, the measure of annual drinking frequency consisted of response categories that comprised any consumption during the last year, i.e., from "monthly or less" to "four or more times a week". Annual drinking frequency was treated as a categorical variable with four levels in correlation and regression analyses, and was collapsed into two categories (frequent/infrequent drinking) for crosstabulation. Frequent drinking consisted of the responses "2–3 times a week" and "≥4 times a week", while infrequent drinking included the response categories "monthly or less" and "2–4 times a month". Binge drinking episodes (AUDIT-3) were measured with the question: "How often, during the last year, did you have six or more drinks on one occasion?". The question was rated on a five-point Likert scale, ranging from "never", "less than monthly", "monthly" and "weekly" to "almost daily". Binge drinking was entered as a categorical variable with five levels in correlation and regression analyses, and was collapsed into two categories (recurrent/never or rarely) for crosstabulation. Recurrent binge drinking included the response categories "monthly", "weekly" and "almost daily", while the responses "never" and "rarely" were combined into a never/rarely caregory. The AUDIT has demonstrated satisfactory psychometric properties and is a recommended alcohol screening instrument [25, 26].

### Measures of sickness presenteeism and impaired daily activities

Sickness presenteeism and impaired daily activities were measured by one item each taken from a Norwegian translation of the Work Productivity and Activity Impairment questionnaire (WPAI). Sickness presenteeism was measured on a visual analogue scale ranging from zero (no influence on productivity) to ten (obstructed productivity completely), where respondents answered the following question: "During the past seven days, how much did alcohol consumption affect your productivity while you were working?". The WPAI has demonstrated satisfactory psychometric properties [27] and measures work productivity in a manner that is in accordance with measures of sickness presenteeism [28], and not only productivity loss in general. Sickness presenteeism was thus found to be a good concept in the context of the present study.

Similarly, impaired daily activities were measured by asking respondents: "During the past seven days, how much did alcohol consumption affect your ability to do regular daily activities, other than work at a job?". Responses were given on a visual analogue scale from zero (no influence on activities) to ten (obstructed activities completely).

Sickness presenteeism and impaired daily activities were entered as continuous variables in correlation and regression analyses, and collapsed into two categories (impairment/no impairment) for utilization in crosstabulation. No impairment reflected a score of zero, while impairment included scores ranging from one to ten on the visual analogue scale.

### Control measures

Earlier studies have found variables such as gender, age, educational level and family life to be associated with activity performance in working populations [29, 30]. Therefore, gender, age, educational level and living status (living alone or living with others) were considered potential confounders and accordingly included as control variables. Additionally, employment sector (private/public) was included as a control measure.

### Analysis

All statistical analyses were performed with IBM SPSS version 24. Bivariate correlation analyses (Pearson *r*) were performed to explore the strength and direction of the unadjusted relationships between the variables. Contingency tables were constructed to estimate the odds and risks of impairment given low or high levels of annual drinking frequency and binge drinking, respectively. Adjusted multiple hierarchical regression analyses were applied to investigate the influence of annual drinking frequency and binge drinking episodes on sickness presenteeism and impaired daily activities. Control measures were entered at stage 1 and alcohol measures were entered in stage 2 to evaluate the model as a whole, as well as the influence of each independent variable. Significant results were defined as *p* < .05.

### Ethics

The study was approved by the Regional Committees for Medical and Health Research in Norway (approval no. 2014/647). Respondents were informed about the study's aim and confidentiality, assured that participation was voluntary and provided written informed consent.

## Results

### Correlations between the variables

As seen in Table 2, correlations between the study variables were generally small, but most were statistically significant.

**Table 2:** Correlations between the study variables.

	**Presenteeism**	**Daily activ.**	**Frequency**	**Binge**	**Gender**	**Age**	**Education**	**Sector**	**Living status**
**Presenteeism**	-								
**Daily activ.**	.712***	-							
**Frequency**	.049**	.107***	-						
**Binge**	.076***	.177***	.341***	-					
**Gender**	-.037*	-.080***	-.109***	-.210***	-				
**Age**	-.029	-.069***	.177***	.,203***	-.051**	-			
**Education**	.019	.023	.131***	-.074***	.023	-.067***	-		
**Sector**	-.031	-.053**	.020	-.139***	.217***	.084***	.300***	-	
**Living status**	-.014	-.051**	.020	-.055**	-.007	-.006	.029	.006	-

Sickness presenteeism and impaired daily activities: Higher scores indicate higher levels of impairment. Gender: Lower score is male, higher score is female; Sector: Lower score is private, higher score is public; Living status: Lower score is living alone, higher score is living with others; For all other variables, higher scores indicate higher levels.

*p < .05

** *p* < .01

*** *p* < .001

### Drinking frequency and binge drinking

Almost two out of ten (19.7%) employees reported “frequent drinking” during last year, i.e., consumption on a weekly or almost daily basis, while the majority (80.3%) reported “infrequent drinking” (maximum four times a month). Approximately one out of ten (11.0%) employees reported “recurrent binge drinking” during the last year (binge drinking episodes on a monthly, weekly or almost daily basis), while 89.0% reported “never or rarely binge drinking”.

As seen in Table 3, 4.2% of employees who consumed alcohol monthly or less reported sickness presenteeism, compared to 7.4% among those who consumed alcohol 2–4 times a month, 9.7% among those who drank 2–3 times a week, and 12.9% among those who consumed alcohol ≥4 times a week. Thus, a higher proportion of frequent drinkers (consumption on a weekly or almost daily basis;10.1%) reported sickness presenteeism compared to infrequent drinkers (consumption maximum 4 times a month; 5.8%). 5.1% of employees who consumed alcohol monthly or less reported impaired daily activities, compared to 11.0% of those who consumed alcohol 2–4 times a month, 16.6% among those who drank 2–3 times a week, and 18.8% among those who consumed alcohol ≤4 times a week. Hence, compared to infrequent drinkers, a higher percentage of frequent drinkers reported impaired daily activities (16.9% versus 8.1%). The odds of sickness presenteeism for frequent drinkers were 1.81 times higher than for infrequent drinkers, while the odds of impaired daily activities for frequent drinkers were 2.32 times higher than for their infrequent counterparts.

**Table 3:** Crosstabulation of annual drinking frequency and activity performance.

	**Drinking frequency**							
	**Monthly or less**		**2–4 times a month**		**2–3 times a week**		**≥4 times a week**	
	**n**	**%**	**n**	**%**	**n**	**%**	**n**	**%**
**Presenteeism**								
Impairment	53	4.2	101	7.4	54	9.7	11	12.9
No impairment	1212	95.8	1268	92.6	505	90.3	74	87.1
**Daily activities**								
Impairment	64	5.1	150	11.0	93	16.6	16	18.8
No impairment	1201	94.9	1219	89.0	466	83.4	69	81.2
**Total n (%)**	1265 (38.6)		1369 (41.8)		559 (17.1)		85 (2.6)	
	**Frequent**^a^				**Infrequent**^b^			
	**n**	**%**	**OR**	**RR**	**n**	**%**	**Total n (%)**	
**Presenteeism**								
Impairment	65	10.1	1.81	1.71	154	5.8	219 (6.7)	
No impairment	579	89.9			2480	94.2	3059 (93.3)	
**Daily activities**								
Impairment	109	16.9	2.32	2.09	214	8.1	323 (9.9)	
No impairment	535	83.1			2420	91.9	2955 (90.1)	
**Total n (%)**	644 (19.7)				2634 (80.3)			

^a^Consumption on a weekly or almost daily basis.

^b^Consumption maximum 4 times a month.

As shown in Table 4, 5.3% of employees who had no binge drinking episodes reported sickness presenteeism, compared to 6.9% among those who rarely binge drank, 8.6% among those who binge drank on a monthly basis, and 30.4% among those who had binge drinking episodes on a weekly basis. Consequently, a higher proportion of recurrent binge drinkers (binge drinking on a monthly, weekly or almost daily basis) reported sickness presenteeism (9.9%) compared to those who never or rarely had binge drinking episodes (6.3%). 5.9% of employees who had no binge drinking episodes reported impaired daily activities, compared to 9.4% among those who rarely binge drank, 24.3% among those who binge drank on a monthly basis, and 34.8% of those who had binge drinking episodes on a weekly basis. Hence, impaired daily activities was indicated by a higher percentage of recurrent binge drinkers (24.9%) than by those who never or rarely had binge drinking episodes (8.0%). The odds of sickness presenteeism for recurrent binge drinkers were 1.64 times higher than for those who never or rarely had binge drinking episodes, while the odds of impaired daily activities were 3.81 times higher for recurrent compared to those who never or rarely had binge drinking episodes.

**Table 4:** Crosstabulation of binge drinking and activity performance.

	**Binge drinking episodes**									
	**Never**		**Rarely**		**Monthly**		**Weekly**		**Almost daily**	
	n	%	n	%	n	%	n	%	n	%
**Presenteeism**										
Impairment	63	5.3	120	6.9	29	8.6	7	30.4	0	0.0
No impairment	1123	94.7	1610	93.1	308	91.4	16	69.6	2	100.0
**Daily activities**										
Impairment	70	5.9	163	9.4	82	24.3	8	34.8	0	0.0
No impairment	1116	94.1	1567	90.6	255	75.7	15	65.2	2	100.0
**Total n (%)**	1186 (36.2)		1730 (52.8)		337 (10.3)		23 (0.7)		2 (0.1)	
	**Recurrent**^a^				**Never/ rarely**^b^					
	**n**	**%**	**OR**	**RR**	**n**	**%**	**Total n (%)**			
**Presenteeism**										
Impairment	36	9.9	1.64	1.59	183	6.3	219 (6.7)			
No impairment	326	90.1			2733	93.7	3059 (93.3)			
**Daily activities**										
Impairment	90	24.9	3.81	3.11	233	8.0	323 (9.9)			
No impairment	272	75.1			2683	92.0	2955 (90.1)			
**Total n (%)**	362 (11.0)				2916 (89.0)					

^a^Binge drinking episodes on a monthly, weekly or almost daily basis.

^b^Never or rarely binge drinking episodes

### Sickness presenteeism

The sickness presenteeism hierarchical regression model is presented in Table 5. The overall model explained 0.8% of the variance in sickness presenteeism. The control variables (gender, age, educational level, living status and employment sector), entered at stage 1, explained 0.4% of the variance in the model. After entering the alcohol consumption variables at stage 2, the total variance explained by the model increased to 0.8%, *F* (7, 3270 = 5.926, *p* < .001), Δ*R*^2^ = .005, *p <* .001. In the fully adjusted model, binge drinking was the only independent predictor associated with sickness presenteeism (b = .040, β = .057, *p* < .01, 95% CI = [.012, .067]). Annual drinking frequency did not display a statistically significant contribution to the model (b = .016, β = .028, *p* = .156, 95% CI = [-.006, .039]).

**Table 5:** Sickness presenteeism hierarchical regression model.

				95% CI	
Variable	b	*SE*	β	Lower	Upper
**Stage 1**					
Gender	-.033	.018	-.033	-.068	.002
Age	-.001	.001	-.026	-.003	.000
Educational level	.015	.010	.028	-.005	.035
Sector	-.046	.029	-.030	-.103	.011
Living status	-.020	.024	-.015	-.066	.026
*R*^2^	.004				
**Stage 2**					
Gender	-.018	.018	-.019	-.054	.017
Age	-.001	.001	-.019	-.002	.001
Educational level	.015	.010	.027	-.005	.035
Sector	-.040	.029	-.026	-.097	.017
Living status	-.016	.024	-.012	-.062	.030
Drinking frequency	.016	.011	.028	-.006	.039
Binge drinking	.040**	.014	.057**	.012	.067
*R*^2^	.008				
Δ*R*^2^	.005***				

**p <. 01

*** *p* < .001

### Impaired daily activities

The impaired daily activities hierarchical regression model is presented in Table 6. The overall model explained 4.2% of the variance in impaired daily activities. The control variables, entered, at stage 1 explained 1.7% of the variance in the model. By including the alcohol measures, the total variance explained increased significantly to 4.2%, *F* (7, 3270 = 50.645, *p* < .001), Δ*R*^2^ = .025, *p* < .001. After controlling for gender, age, educational level, employment sector and living status, both annual drinking frequency and binge drinking were significantly associated with impaired daily activities. Binge drinking (b = .120, β = .131, *p* < .001, 95% CI = [.085, .155]) displayed a stronger influence on daily activity impairment than annual drinking frequency (b = .049, β = .064, *p* < .01, 95% CI = [.020, .078]).

**Table 6:** Impaired daily activities hierarchical regression model.

				95% CI	
Variable	b	*SE*	β	Lower	Upper
**Stage 1**					
Gender	-.098***	.023	-.076***	-.143	-.053
Age	-.004***	.001	-.068***	-.006	-.002
Educational level	.025	.013	.034	-.001	.050
Sector	-.083*	.038	-.041*	-.158	-.009
Living status	-.094**	.031	-.053**	-.154	-.034
*R*^2^	.017				
**Stage 2**					
Gender	-.055*	.023	-.042*	-.101	-.009
Age	-.003*	.001	-.052*	-.005	-.001
Educational level	.024	.013	.033	-.002	.050
Sector	-.066	.038	-.032	-.139	.008
Living status	-.083**	.030	-.047**	-.142	-.023
Drinking frequency	.049**	.015	.064**	.020	.078
Binge drinking	.120***	.018	.131***	.085	.155
*R*^2^	.042				
Δ*R*^2^	.025***				

*p < .05

**p < .01

***p < .001

## Discussion

The aim of the present study was to explore the influence of annual drinking frequency and binge drinking on activity impairments both at work (sickness presenteeism) and outside the workplace. Results showed that (a) binge drinking was associated with higher levels of sickness presenteeism and impaired daily activities, (b) binge drinking had a stronger influence on daily activities than on sickness presenteeism, and (c) annual drinking frequency significantly influenced the employees’ daily activities but it did not affect sickness presenteeism.

Binge drinking was associated with both higher levels of sickness presenteeism and impaired daily activities outside the workplace. Binge drinking is known to have several short-term effects such as hangovers, decreased attention and reduced concentration, as well as other temporary physical, cognitive and psychological disturbances [31]. These consequences can severely impact the individual's ability to perform regular daily activities and reduce their work performance [32, 33]. Reduced on-the-job performance due to alcohol consumption seems to be fairly common amongst the workforce, and the findings from this study are comparable to other studies on the Norwegian working community [23].

Somewhat surprisingly, the association between binge drinking and impaired daily activities was stronger the association between binge drinking and sickness presenteeism. Similarly, annual drinking frequency displayed an influence on impaired daily activities but not on sickness presenteeism. An explanation for these findings could be that (heavy) drinking usually occurs on days preceding weekends and holidays, when the employees have a day off from work [34].

Studies on drinking patterns have found that people drink less before conducting "serious" activities that require long-term commitment and focus, such as work activities, due to the impact heavy drinking can have on performance [35]. Another related explanation could therefore be that the employees moderate their behavior because of a fear of sanctions as a consequence of reduced performance due to alcohol. In Norway, alcohol in the workplace is considered inappropriate [36]. Behavior that deviates from these norms may lead to marginalization, social exclusion [37], formal admonitions from employers and in some cases even resignation [22]. It is therefore possible that fear of such sanctions might contribute to self-regulation and suppression of impairments while at work, whereas similar self-regulation is not considered necessary outside the workplace. These findings seem to be in line with Frone's [3] general model of employee substance use and productivity outcomes that postulates that reduced on-the-job productivity primarily is a result of on-the-job substance use.

By comparing standardized regression coefficients and probability values, the present study found that annual drinking frequency had less influence on both activity performance measures compared to binge drinking. It is possible that, whereas binge drinking episodes result in more short-term disability and impairments, a pattern of frequent consumption can have more long-term consequences which do not immediately influence employees' activity performance in a short-term perspective [16]. Individuals who have a pattern of frequent drinking often experience more serious health-related problems in the long-term [38], and it is therefore likely that frequent drinkers might have more sickness absence compared to employees who engage in infrequent binge drinking. Research on the relationship between alcohol consumption and sickness absence has found that a larger number of drinks consumed per week is associated with a higher number of sickness absence days during a year [39]. Employees who drink frequently do not necessarily consume large amounts of alcohol on each occasion. Hangovers and other impairments due to alcohol usually result from episodes of heavy consumption, whereas low-risk drinking is not associated with next-day impairments [40].

### Implications

Findings from the present study might indicate that binge drinking has a stronger influence on activity performance than annual drinking frequency, both at work and outside the workplace. Hence, individual and collective interventions aimed at preventing the development of alcohol-related problems may benefit from specifically targeting alcohol consumption behavior characterized by high levels of binge drinking. The findings from this study may in particular have implications for public sector employees, as a result of well educated female employees above age 40 and employed within public administration constituting a large proportion of the study sample.

### Methodological issues

The present study has some limitations. It was based on a cross-sectional design and, hence, it is not possible to draw causal inferences from the associations identified. The relationship between alcohol consumption and activity performance may, as emphasized by Frone [3], be moderated and influenced by a variety of variables not included in the present study, such as various pharmacological, dispositional, situational and motivational factors. Such presumed complexity may be a pivotal reason for why the present study's included variables were not able to explain a large proportion of variance in the outcome measures.

This study was based on a relatively large sample (n = 3,278). The final response rate, however, was low (22.8%). Moreover, comparisons between our study sample and characteristics of the entire Norwegian workforce did reveal that older, highly educated and female employees were somewhat overrepresented in this study. On the other hand, our study sample was to a much lesser degree different from our invited sample regarding gender and age distributions. Gender distribution in the study was not significantly different from the invited sample. Age distribution, however, was significantly different (*p* < .001), with a 4.0% underrepresentation of employees younger than 40 years old. Although non-response is a less prominent threat to associations between variables than to prevalence estimates [41], the low response rate may have somewhat biased our findings. Some studies suggest that males, individuals with low socioeconomic status and heavy drinkers tend to be underrepresented in health surveys [41–43]. Furthermore, actual alcohol sales have been found to be considerably higher than self-reported alcohol consumption [44]. Non-response bias and the application of self-reported alcohol measures suggest that alcohol consumption may be underestimated in this study. As such, findings must be interpreted with some caution.

We measured our four main variables with only one item on each, which could be a limitation in how we were able to grasp the concept under study. However, all four items were taken from validated instruments using psychometric accepted scales, and single-item measurements have been demonstrated to be reliable when exploring health behaviors, especially when inquiring about rather objective facts [45]. Our independent and dependent variables were measured within different time frames, i.e., consumption during the last year and impairment during the last seven days. Measuring consumption within a large time frame may have rendered it possible to capture a presumably representative drinking pattern, although it may have increased the risk for recall bias. Conversely, the activity performance measures may have had a limited ability to grasp a representative impairment pattern due to the restricted time frame, although minimizing the risk for recall bias.

We chose to interpret work productivity as sickness presenteeism, even though we are aware of the differing opinions on how presenteeism should or could be measured. Some argue that combining "showing up at work feeling ill" with "productivity loss" provides a complex outcome element that is both difficult to define and to measure. Therefore, some propose that presenteeism should only involve "showing up for work when one is ill" [46]. Given the employers' perspective and the socioeconomic perspectives on presenteeism, it may be conversely claimed that it is when this situation results in productivity loss that it becomes of interest. Being at work, not feeling well, but performing as normal is a phenomenon with less impact. Believing that all who feel unwell will have reduced productivity may involve overestimating the effect of illness. Therefore, in this study presenteeism is clearly linked to the consequences of alcohol use on illness and productivity. Furthermore, in this study we conceptualized frequent drinking as consuming alcohol at least two times a week, while recurrent binge drinking was operationalized as binge drinking episodes occurring on a monthly basis or more. These thresholds were chosen to reflect the dry drinking culture in Norway, a culture characterized by binge drinking during the weekends and abstinence during weekdays [22]. What constitute appropriate cut-off values may vary considerably between countries and cultures [47].

Our outcome measures did not allow us to estimate the number of lost hours or days of productivity associated with increased alcohol consumption. However, the aim of the present study was not to provide such estimations but rather to compare the relative influences of two alcohol measures on two activity performance arenas. The wording of the WPAI-statements may be considered to measure a relationship as well as a construct, e.g., by asking respondents to indicate whether they have experienced productivity loss due to alcohol consumption. Hence, participants are asked to attribute their behavior to a specific cause, and such attributions may not be accurate. However, the WPAI is considered to be a valid instrument [20] and was, despite some inherent limitations, deemed serviceable in the context of this study.

## Conclusions

Alcohol consumption constitutes a global health issue. The present study found that employees' alcohol consumption were associated with their activity performance both at work (sickness presenteeism) and outside the workplace. Binge drinking was stronger associated with activity impairments than annual drinking frequency, and binge drinking was stronger associated with daily activities than with workplace performance. Although further longitudinal research is needed, the findings of the present study implicate that interventions targeting alcohol consumption should place large emphasis on binge drinking behavior.
